# Supplementary material for: Association between oral microbiome and five types of respiratory infections: a two-sample Mendelian randomization study in east Asian population
Source: Front Microbiol. 2024 Apr 10;15:1392473. doi: 10.3389/fmicb.2024.1392473 (PMC11039966; doi:10.3389/fmicb.2024.1392473)
Supplement: Supplementary file 1 [file Data_Sheet_1.zip › Supplementary Materials/MR plots of saliva/Tonsillitis/g__Eggerthia/leave_one_out.pdf]

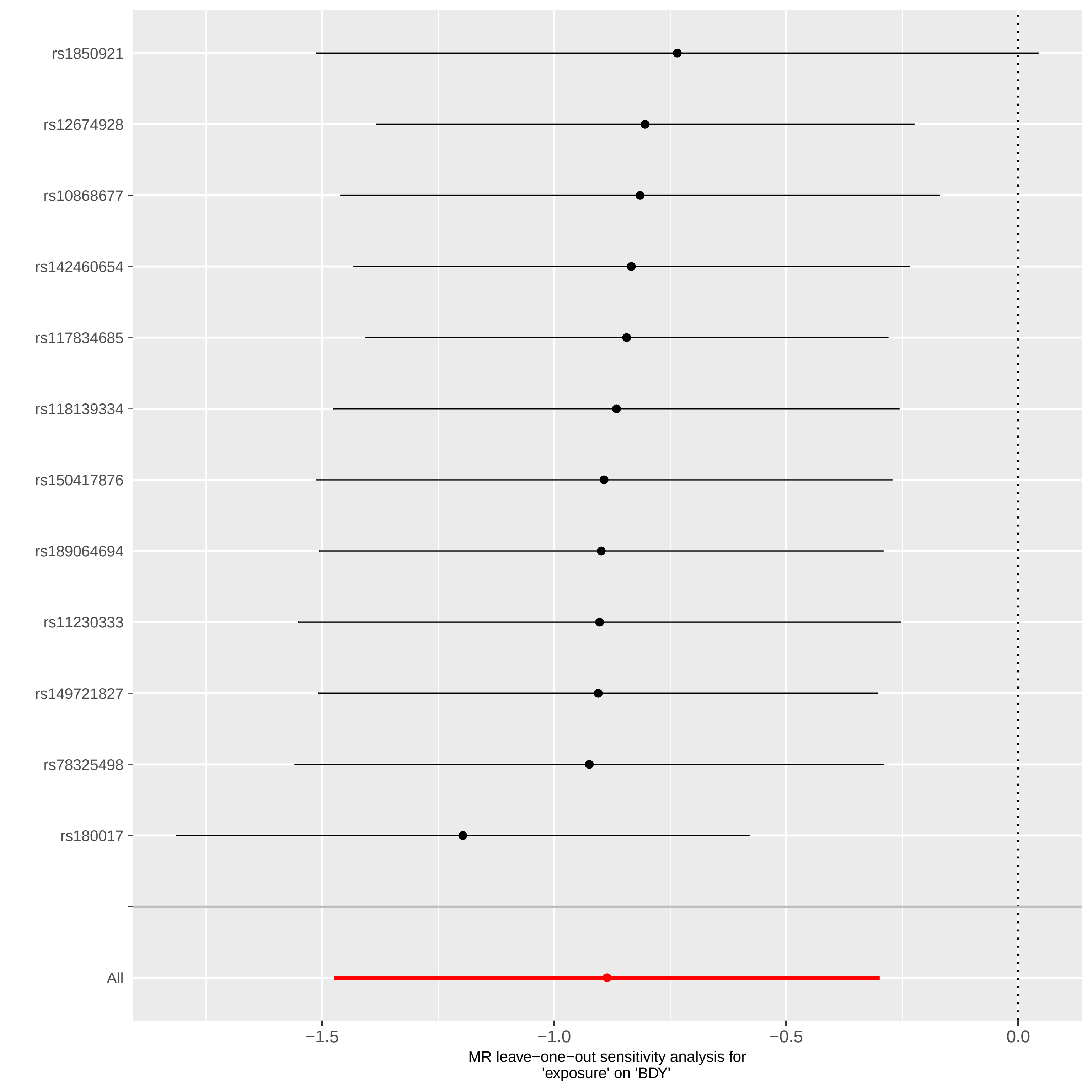
rs1850921
rs12674928
rs10868677
rs142460654
rs117834685
rs118139334
rs150417876
rs189064694
rs11230333
rs149721827
rs78325498
rs180017
All
−1.5
−1.0
−0.5
0.0
MR leave−one−out sensitivity analysis for
'exposure' on 'BDY'
